# Repeated stressors in adulthood increase the rate of biological ageing

**DOI:** 10.1186/s12983-015-0095-z

**Published:** 2015-02-13

**Authors:** Michaela Hau, Mark F Haussmann, Timothy J Greives, Christa Matlack, David Costantini, Michael Quetting, James S Adelman, Ana Catarina Miranda, Jesko Partecke

**Affiliations:** Max Planck Institute for Ornithology, Evolutionary Physiology Group, Am Obstberg 1, 78315 Radolfzell, Germany; Department of Biology, University of Konstanz, Universitätsstraße 10, 78464 Konstanz, Germany; Department of Biological Sciences, North Dakota State University, 1340 Bolley Drive, Fargo, ND 58202 USA; Department of Biology, Bucknell University, Lewisburg, PA 17837 USA; Department of Biology, University of Antwerp, Universiteitsplein 1, 2610 Wilrijk, Belgium; University of Glasgow, Institute of Biodiversity, Animal Health & Comparative Medicine, Glasgow, QG12 8Q UK; Department of Biological Sciences, 4092B Derring Hall, Virginia Tech, Blacksburg, VA 24061-0406 USA; Max Planck Institute for Ornithology, Department of Migration and Immuno-ecology, Am Obstberg 1, 78315 Radolfzell, Germany; Departamento de Biologia, Centro de Ciências Biológicas e da Saúde, Universidade Federal do Maranhão, Campus do Bacanga, São Luís, Maranhão Brazil

**Keywords:** Biomarker, Repeated stressors, Eurasian blackbird, Oxidative stress, Glucocorticoid, Telomere

## Abstract

**Background:**

Individuals of the same age can differ substantially in the degree to which they have accumulated tissue damage, akin to bodily wear and tear, from past experiences. This accumulated tissue damage reflects the individual’s biological age and may better predict physiological and behavioural performance than the individual‘s chronological age. However, at present it remains unclear how to reliably assess biological age in individual wild vertebrates.

**Methods:**

We exposed hand-raised adult Eurasian blackbirds (*Turdus merula*) to a combination of repeated immune and disturbance stressors for over one year to determine the effects of chronic stress on potential biomarkers of biological ageing including telomere shortening, oxidative stress load, and glucocorticoid hormones. We also assessed general measures of individual condition including body mass and locomotor activity.

**Results:**

By the end of the experiment, stress-exposed birds showed greater decreases in telomere lengths. Stress-exposed birds also maintained higher circulating levels of oxidative damage compared with control birds. Other potential biomarkers such as concentrations of antioxidants and glucocorticoid hormone traits showed greater resilience and did not differ significantly between treatment groups.

**Conclusions:**

The current data demonstrate that repeated exposure to experimental stressors affects the rate of biological ageing in adult Eurasian blackbirds. Both telomeres and oxidative damage were affected by repeated stress exposure and thus can serve as blood-derived biomarkers of biological ageing.

**Electronic supplementary material:**

The online version of this article (doi:10.1186/s12983-015-0095-z) contains supplementary material, which is available to authorized users.

## Introduction

Within populations of wild animals, individuals of the same age class can show vast variation in physiological and behavioural performance, as seen in self-maintenance processes like immune function and DNA repair or the degree of reproductive investment [1]. Understanding the causes and consequences of individual variation in behaviour and physiology has long been a subject of intensive research [2], and it has also permeated into the demographic and medical fields [3,4]. Variation among individuals can arise from differences in genetic quality and conditions during development, but also from both past and current health status and stressful experiences that may have caused cellular damage akin to bodily wear and tear. The extent of cellular damage that an individual has accumulated can affect physiological and behavioural traits through effects on physical condition, resource allocation and mortality risk [4-13]. However, although scientific and popular interest in the genetic, physiological and evolutionary factors underlying an individual’s biological age has intensified [14-20], it is still unclear how it can be reliably assessed in wild vertebrates [20-24].

The mechanisms that underlie ageing (or senescence) are currently under lively debate [16,18,23,25,26]. One predominant concept is the oxidative stress theory of ageing, which assumes that cells continuously generate endogenous oxygen radicals which, especially when produced at elevated rates, damage essential molecules such as DNA, lipids and proteins [10,14,27-29]. Another popular concept focuses on the attrition of telomeres [30-34]. Telomeres are repetitive sequences of DNA at the ends of eukaryote chromosomes that shorten with each replication event and when reaching a critical length, induce a permanent arrest in the cell cycle [35,36]. The oxidative stress theory of ageing and the telomere attrition theory are mechanistically linked since telomere shortening can be exacerbated by free radical attack [37]. A third group of hypotheses, the allostatic load/reactive scope theories suggest that the adaptive function of the catecholamine and glucocorticoid hormone systems to cope with disturbances can turn pathophysiological when becoming chronically over-activated in stressed individuals [38-41].

While these theories are not mutually exclusive and their proposed mechanisms likely interact in complex ways [21,22], they suggest potential blood-derived biomarkers that could serve as indicators of individual health and cumulative damage in wild populations: measures of oxidative stress load, telomere dynamics, and circulating concentrations of glucocorticoid hormones. While progress is being made in understanding the links between these markers and biological ageing, most studies have been limited by concentrating on single variables or certain physiological systems, or by collecting data cross-sectionally on a population level. Furthermore, to our knowledge most work on how stressful experiences in adulthood affect biomarkers of health and ageing has been conducted in humans, using correlative rather than experimental approaches [42]. In non-human vertebrates experimental studies are increasingly being carried out, but so far have mostly focused on the effects of stressful experiences early in life (e.g. [43-46]).

**Figure 1:**
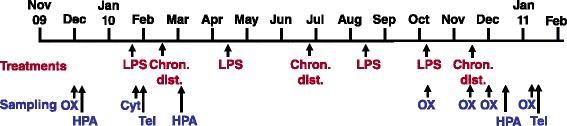
**Timeline of experiment.** Treatment types: LPS: LPS injections, Chron. dist.: chronic disturbance. Biomarker sampling: Tel: telomeres, OX: oxidative stress, HPA: glucocorticoid hormones, CYT: cytokines.

To improve our understanding of the factors that allow the determination of an individual’s accumulated tissue damage and thus its biological age, we conducted an experimental study under common garden conditions on adult Eurasian blackbirds (*Turdus merula*). To generate differences in wear-and-tear among adult individuals with similar ontogenetic experiences (hand-raised birds kept under standardised conditions from early age onward), we exposed one group of birds to repeated immune and disturbance challenges, while a control group experienced identical conditions but was not subject to these stressors (Figure 1). Because we did not know the extent of accumulated tissue damage for each individual before we began our study, and since organisms in nature are exposed to a variety of stressors, we chose to apply a combination of the two stressors. In addition, based on the likely connections among the oxidative stress, telomere, and allostatic load theories [31], a particular strength of our approach was to explore all three of these potential mediators of biological ageing in concert (Figure 1).

Based on the oxidative stress, telomere, and allostatic load theories and the known relationships among their peripheral markers, we made the following predictions for the outcome of our experiment. First, we expected changes in general indicators of individual condition over time, such as a decrease in body mass [47] and an increase in locomotor activity [48]. These two measures are commonly used to provide an easy assessment of short-term changes in individual condition. Second, we expected that individuals from our stress-exposed group would have more telomere shortening than control birds by the end of the experiment. We chose telomere length as a likely endpoint, because studies in several vertebrate species including humans have linked both absolute telomere length as well as telomere shortening rates to individual life span and disease risk [5,7-9,11,12,49-51]. The impact of stressors on telomeres is thought to be mediated by pathways involving both glucocorticoid hormones and oxidative stress [13,26,28,31,46,49,52-55]. Glucocorticoid hormones may also increase oxidative stress and thereby further contribute to telomere shortening rates [46]. Therefore, since both circulating baseline and stress-induced glucocorticoid concentrations have been used traditionally to assess an individual’s responses to stressors [38,56,57], we thirdly predicted that individuals from our stress-exposed group would show increased concentrations of baseline corticosterone (the main glucocorticoid in birds), muted peak corticosterone secretion after disturbance, and a delayed negative feedback (shut-down of corticosterone secretion once a stressor has abated). Such traits are typically considered hallmarks of chronically stressed individuals (e.g., [38,56,57], but see [47]). Fourth, we expected stress-exposed individuals to display a greater oxidative stress load as oxidative stress can increase following demanding experiences such as high physical activity [7,27,58], immune challenges [59-61], egg production [46,62], or embryonic exposure to stress hormones [46]. Oxidative stress is thought to affect telomere dynamics on the one hand by directly targeting telomeric DNA, which is particularly vulnerable to oxidative damage [37,63], and on the other hand by damaging telomerase, the enzyme that can rebuild telomeres [24,31,37]. We further predicted that stress-exposed birds will up-regulate antioxidant defences compared to control birds. An up-regulation of antioxidant defences may also only occur initially; as long-term stress exposure may eventually overwhelm self-maintenance systems.

## Results

**Figure 2:**
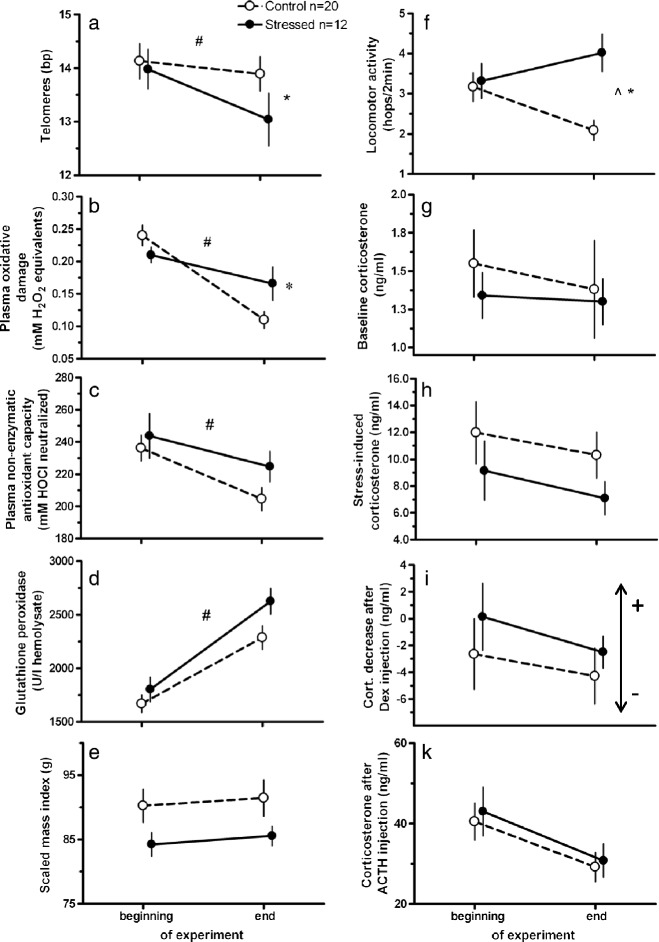
**Long-term effects of treatment on potential biomarkers. (a)** Telomere length, **(b)** plasma oxidative damage, **(c)** plasma non-enzymatic antioxidant capacity, **(d)** red blood cell glutathione peroxidase, **(e)** Scaled Mass Index, **(f)** locomotor activity, and plasma concentrations of **(g)** baseline corticosterone, **(h)** stress-induced corticosterone, **(i)** difference in corticosterone between stress-induced levels and concentrations after injection of dexamethasone (please note that positive values indicate strong (+) negative feedback whereas negative values show weak (−) negative feedback , see also arrow), **(k)** corticosterone after injection of ACTH. Open circles: control group (n = 20), filled circles: stress-exposed group (n = 12). All data are mean ± 1SEM. Results as derived from LMMs (see text): * interaction time*treament p < 0.05, ^ treatment p < 0.05, # time p < 0.05.

As a long-term consequence of the treatment, telomere dynamics of birds from the two groups diverged: while birds from both treatment groups decreased genome-wide telomere lengths, stress-exposed birds showed a stronger decrease in telomere lengths from the beginning to the end of the experiment than control birds (Figure 2a; all statistical results including notes on transformations are presented in Table 1). All birds decreased circulating oxidative damage over time (Figure 2b), but stress-exposed birds showed a less robust decrease than control birds, thus maintaining comparatively higher levels of oxidative damage. Plasma non-enzymatic antioxidants decreased (Figure 2c) and glutathione peroxidase concentrations in red blood cells increased over time (Figure 2d), but there were no differences between groups, respectively. All birds tended to increase body condition over time, but there were no group differences (Figure 2e). However, locomotor activity in the two groups was differentially affected by treatment: control birds decreased while stress-exposed birds maintained or slightly increased their activity rates (Figure 2f). We did not detect changes over time or differences between groups in any measure of the endocrine stress axis (Figure 2g-k).

**Table 1:** **Effects of treatment on potential biomarkers**

	**Effect of treatment**	**Effect of time**	Interaction treatment*time
Genome-wide telomere length (bp)	p > 0.28	**F**_**(1,32)**_ **= 28.81, p < 0.0005**	**F**_**(1,32)**_ **= 11.47, p = 0.002**
Plasma oxidative damage (mM H_2_O_2_ equivalents)	p > 0.2	**F**_**(1,30.99)**_ **= 42.46, p < 0.0005**	**F**_**(1, 30.99)**_ **= 6.84, p = 0.014**
Plasma non-enzymatic antioxidants (mM HOCl neutralized)	p > 0.1	**F**_**(1,29.75)**_ **= 8.49, p = 0.007**	p > 0.3
Glutathione peroxidase (U/l hemolysate)	p > 0.1	**F**_**(1,30.71)**_ **= 64.54, p < 0.0005**	p > 0.26
Scaled mass index	p > 0.12	F_(1,32)_ = 3.65, p = 0.065	p > 0.7
Locomotor activity (hops/min.)	**F**_**(1,32)**_ **= 5.18, p = 0.03**	p > 0.1	**F**_**(1,32)**_ **= 21.84, p < 0.0005**
Baseline corticosterone (ng/ml)	p > 0.7	p > 0.3	p > 0.5
Stress-induced corticosterone (ng/ml)	p > 0.3	p > 0.4	p > 0.8
Difference between stress-induced corticosterone and concentrations after dexamethasone injection (ng/ml)	p > 0.1	p > 0.6	p > 0.7
Corticosterone after ACTH injection (ng/ml)	p > 0.5	p > 0.09	p > 0.6

Effects of stress-exposure (repeated LPS injections and 10-day chronic disturbance protocols combined) on potential biomarkers (all log-transformed) assessed at the beginning and the end of the experiment, as determined from LMM statistics (for details see text). Significant effects are highlighted in bold. Data are shown in Figure 2.

## Discussion

More rapid decreases in telomere lengths generally indicate a more rapid rate of cellular ageing [30-34]. We found that chronic stress exposure in hand-raised Eurasian blackbirds resulted in greater telomere loss compared to control birds, providing experimental evidence that one outcome of stress exposure in adult blackbirds is an increase in cumulative cellular damage. While we were not able to follow the survival of our experimental birds, it is possible that our stress-exposed blackbirds could have suffered a greater mortality risk. Indeed, previous studies on vertebrate species including humans have suggested that individuals with shorter telomeres or an increased rate of telomere shortening have shorter life spans [5,7-9,11,12,49-51]. Importantly, while a growing number of studies suggest that early life stress can hasten telomere loss [43-46], our study is one of the first experimental tests indicating that stress in adulthood results in more rapid telomere loss and an increase in cellular ageing.

Over the course of the current experiment, stress-exposed birds maintained higher levels of plasma oxidative damage than control birds (Figure 2; Table 1). This corroborates previous findings in which relationships between immune challenges, psychological stress and oxidative damage have been demonstrated [10,28,59-61]. We can currently only speculate as to why there was a general decrease in plasma levels of oxidative compounds over time in both groups. It is possible that this decrease is the result of prolonged captivity (in control birds it was paralleled by a decrease in locomotor activity). Nevertheless, stress-exposed birds showed a significantly smaller decrease, thus ending up with a higher level of circulating oxidative compounds than control birds at the end of the experiment.

We determined circulating concentrations of oxidative damage and non-enzymatic antioxidants from plasma while glutathione peroxidase and telomere measures were obtained from red blood cells. By determining biomarkers from blood samples we aimed at quantifying systemic effects of the stressors rather than effects on specific tissues. Furthermore, this technique enabled us to repeatedly sample individuals to assess longitudinal effects of stressful experiences. Plasma and red blood cells are both components of the same type of connective tissue and preliminary data show a significant covariation of measures derived from blood components with oxidative processes in other tissues [64]. Also, a covariation among oxidative markers of red blood cells and of plasma similar to those used in the present study was demonstrated in another songbird species, although the strength of covariation can change with the intensity of a challenge [58]. Furthermore, telomere lengths in blood cells and other tissues are correlated in humans [65] and birds [66]. Given that both oxidative stress and telomere measures correlate among tissues, we feel justified in using markers in blood (cells and plasma) as proxies for what is happening in the whole organism.

Contrary to our predictions, specific effects of treatment could not be detected in other potential biomarkers like antioxidants, and all four corticosterone traits that we assessed (Figure 2; Table 1). There are a few possible explanations for why our predictions regarding these markers were not supported. One possibility is that the benign conditions in captivity might have mitigated the effects of the stress exposure on these biomarkers. An alternative explanation could involve the limited sample sizes, which could have prevented us from detecting significant group differences. Indeed, a visual examination of Figure 2 suggests that both plasma non-enzymatic antioxidant and red blood cell glutathione peroxidase concentrations tend to be higher in stress-exposed compared to control birds, in line with our prediction of an up-regulation of antioxidant processes in individuals that show higher concentrations of damaging compounds in their plasma. And finally, it is also possible that some biomarkers indeed were not affected much by the stress exposure, either because they do not represent appropriate biomarkers for cumulative tissue damage or because the organism is resilient and can cope with the treatment for these specific traits.

Our analyses of the short-term effects of the two treatment types (Additional file 1: Table S1, Additional file 2: Table S2) indicated that both treatments (immune and disturbance challenges) caused significant group divergences in body mass, in plasma oxidative damage and in cytokines (measured only after immune challenge), but not in antioxidants or endocrine traits (only measured after the chronic disturbance treatment). Interestingly, plasma oxidative damage showed opposite short-term responses in stress-exposed birds, increasing following LPS injection (Additional file 1: Table S1) but decreasing following chronic disturbance (Additional file 2: Table S2). Due to logistical constraints we ended up collecting samples for plasma oxidative damage following the first injection of LPS in January 2010, at the beginning of the experiment (see Figure 1), while plasma oxidative damage following chronic disturbance were assessed during the last chronic disturbance period in November 2010 (Additional file 2: Table S2, Figure 1). While an increase in plasma oxidative damage following LPS injection is expected after an activation of the immune system [59-61], a decrease following a disturbance stressor is not. However, this decrease in plasma oxidative damage in stress-exposed birds is actually the result of elevated levels at the beginning of the chronic disturbance period and a subsequent decrease to the levels that control birds displayed throughout (Additional file 2: Table S2). Hence, part of this pattern might be explained by stress-exposed birds having increased levels of oxidative stress at this late stage of the experiment before the chronic disturbance started. Why these levels then decreased during the 10 days of disturbance stressor is currently unclear, but may be related to increased clearance from the circulation since stress-exposed birds tended to have higher levels of plasma non-enzymatic antioxidants and red blood cell glutathione peroxidase than control birds (Additional file 2: Table S2).

Over the course of the year-long experiment, we also observed changes in general indicators of individual condition. Body mass increased over time, but there were no discernible differences between the two groups. However, stress-exposed birds displayed greater locomotor activity than control birds by the time the experiment was ended (Figure 2; Table 1). Further experiments are needed to determine whether increased activity rates are the end result of exposure to stressors or whether they also directly contributed to increased oxidative damage in the stress-exposed group as previously shown in another song bird species ([58]).

Among the strengths of the present study are the experimental approach but also the repeated assessment of individuals. Our results confirm that repeated sampling of focal individuals is important for interpreting studies on cumulative damage and cellular senescence. In the present study, many of the potential biomarkers assessed showed significant changes over time, even in control individuals (Figure 2). We consider it likely that they changed as part of the natural ageing process since we aimed at sampling the birds at the same time of year at the beginning and the end of the experiment to control for any seasonal variation. However, the repeated measures design allowed us to disentangle effects of time from effects that were specific to treatment.

Analyses of intra-class correlation coefficients (ICCs; [67]) of potential biomarkers can provide a measure of their within-individual repeatability and thus their reliability in indicating an individual’s health. Keeping in mind the limitations of our data set (limited sample sizes and number of repeated measurements), ICC analyses suggested that telomere lengths, scaled mass index and stress-induced corticosterone concentrations were highly repeatable traits of individuals (Additional file 3: Table S3). Plasma oxidative damage and glutathione peroxidase levels also showed significant ICCs, although with lower coefficients. Hence, since the variables telomere length and oxidative damage were the ones that showed significant treatment effects as well as significant repeatability, this suggest that these measures, in particular telomere length, are relevant biomarkers for an individual’s health and senescence status.

## Conclusions

Effects of treatment on telomere dynamics and oxidative damage were observed in adult, known-age individuals held under relatively benign conditions in the laboratory including mild temperatures, absence of predation, and food *ad libitum*. Consequences of exposure to stressors may be more pronounced in free-living individuals that are exposed to an array of more severe challenges and a greater limitation of resources than the captive individuals in our study. Other long-term stressors present in nature, for example social challenges, predation pressure, infections, parasites, as well as anthropogenic disturbances, noise, or pollutants may similarly affect telomere dynamics and oxidative damage, possibly reducing the fitness of individuals in the wild. Experimental stress-exposure resulted in telomere shortening and increased concentrations of plasma oxidative damage, which together with the significant repeatability in these measures, suggest that these traits can serve as biomarkers for health and senescence in individuals of a passerine bird, and possibly other taxa as well. Future research will be important for unravelling the causal connections among the different biomarkers.

## Methods

### Study species

The Eurasian blackbirds used for this experiment were 2.5 years old at the start of the experiment (life span in the wild has been estimated at 1.8-2.8 years, but upon reaching adulthood, Eurasian blackbirds live can live up to 17 years [68]). The birds (sample size: 49) were originally collected in 2007 at an age of 5–11 days from 9 urban and 9 rural nest (see Additional file 4 for details) and hand-raised under identical conditions (see [69]). Here we only analysed data from individuals for which we were able to obtain data for all biomarkers before and after the experiment (n = 32, 20 control [11 male, 9 female], 12 stressed [8 male, 4 female] birds). This conservative approach allowed us to keep the sample size and identity of birds identical across different types of analyses. However, analyses of the entire data set (including individuals for which biomarkers could not be obtained for all time points) gave similar results. Because of the limited sample size and the concomitant low power in complex statistical tests we also refrained from analysing differences in responses to treatment of urban and rural birds (but see [70]).

Birds were kept in individual recording cages and were exposed to a simulated local natural photoperiod in Radolfzell, Germany (see Additional file 4 for details). Before the experiment started we measured tarsus length of all birds as a measure of structural size to the nearest 0.1 mm with a dial calliper and body mass with a digital balance to the nearest 0.1 g. Body mass was assessed repeatedly during the experiment, when we caught the birds for blood sampling. When establishing experimental groups we made sure that sex and origin were balanced among control and stress-exposed groups. The stress-exposed group was exposed to one of two alternating stress treatments about every 6 weeks (for timeline and sequence of treatments see Figure 1): a.) an acute immune challenge (see below; four times over the course of the experiment), and b.) a chronic stress disturbance regime (see below; a total of three times; Figure 1).

### Stress treatments

We injected a dose of 2.0 μg LPS (Sigma L2880) diluted in phosphate-buffered saline (PBS) per gram body mass into the breast muscle (see Additional file 4 for details). Control birds were injected with PBS only (but only for the first two periods when cytokine concentrations after injection were compared between treatment groups). The concentration of LPS used successfully induces an acute phase response including fever and sickness behaviour in other songbird species [71,72]. For verification of LPS injections on cytokine concentrations in this study see Additional file 1: Table S1.

Our chronic stress disturbance regime represented a milder version of existing chronic stress protocols for small passerines (i.e. fewer disturbances per day and fewer days with disturbances than in [73,74]). Every day, for 10 consecutive days, we applied each of the following four treatments to birds in the stressed group, in a random order and at random times, but always during daylight hours: 30 min of chasing (waving a catching net with a yellow plastic bag attached in front of and over the top of the cage for a conspicuous and noisy disturbance), 30 min of crowding (adding 2–3 other birds to a single cage), 60 min of restraint (putting an individual into a cloth bag) and 60 min of loud radio playing in the room. We used this protocol in trying to avoid habituation to a single stressor.

### Biomarkers

In total, stress-exposed birds were subject to 4 immune challenges and 3 chronic disturbance stress periods during the year-long experiment. All control and stress-exposed birds were sampled for biomarkers at the same time periods (see Figure 1). Blood samples were collected from the wing vein in heparinised capillary tubes and stored on ice until centrifugation. The plasma fraction was frozen for later hormone and oxidative stress analyses, the red blood cells either stored in cryoprotectant buffer for telomere analyses or frozen and stored at −80°C for glutathione peroxidase analyses (see Additional file 4 for details and sampling times). To determine the long-term effects of treatment, we measured the oxidative status of plasma and red blood cells at the beginning (Dec 2009) and at the end of the experiment (Jan 2011, Figure 1). To verify the efficacy of our treatments and to confirm that our treatments were capable of inducing changes in oxidative status, blood samples were collected in October 2010 to assess the short-term effects of LPS injection on oxidative status, and in November 2010 samples were collected to assess the short-term effects of the chronic stress protocol.

Plasma oxidative damage was determined using the d-ROMs test (Diacron International, Grosseto, Italy). This assay mostly measures oxidative damage compounds generated early in the oxidative cascade (i.e., hydroperoxides); these compounds are precursors of several end-products of lipid peroxidation, such as malondialdehyde, hydroxynonenal and isoprostanes. The reaction of a dilution series of cumene hydroperoxide with the d-ROM reagents was highly linear (range: 0 to 4.5 μM, *R*^2^ = 0.9996; physiological values in vertebrates). The OXY-Adsorbent test (Diacron International) was used to quantify the ability of plasma non-enzymatic antioxidant compounds (vitamins, carotenoids, uric acid, thiol proteins) to react in vitro with HOCl (oxidant of pathologic relevance in biological systems). The concentration of glutathione peroxidase in red blood cells was quantified using the Ransel assay (Randox Laboratories, Crumlin, UK). For further details on all procedures including assay quality see Additional file 4.

Total locomotor activity of individual birds was recorded continuously and binned in two-minute-intervals using passive infrared motion detectors (see Additional file 4 for more details). For each individual bird, we calculated mean activity over five complete days during the same periods (20-24th Jan) at the beginning (2010) and the end of the experiment (2011). Such averaging of activity rates was done to limit any unexpected differences in circadian variations in locomotor activity among individuals and between groups. Comparing the activity levels between the two groups at the same time of year allowed us to distinguish whether differences in individual activity were due to treatment versus age effects while controlling for possible seasonal changes in activity.

Telomeres were measured with the Telomere Restriction Fragment (TRF) assay and the procedure was carried out on whole blood as in previous studies [46], see Additional file 4 for more details, including assay quality). Leukocytes account for less than 0.2% of the blood volume in birds, and so TRF measurements largely reflect erythrocyte telomeres.

Our protocol for the assessment of the functioning of the endocrine stress axis was modified from existing protocols [73,75,76]. We conducted the stress axis assessment on a subset of individuals from both groups each day, sampling as many individuals as was feasible within three minutes of entering the room. Remaining individuals were sampled on consecutive days in an identical manner, with sample collection of the entire study population being completed within a total of 3–4 days for each sampling period (always changing the sequence of sampled individuals between each sampling period). Samples (100 μl blood) for baseline corticosterone concentrations (BaseCort) were always taken within 3 min of entering the experimental room. Stress-induced samples (100 μl blood, StressCort) were taken after 30 min of restraint in individual cloth bags. Birds then were injected intramuscularly with a dose of 200 μg/kg dexamethasone (DEX; stock solution 4 mg/ml, Bela-Pharm, Germany) dissolved in 0.9% saline (Braun), to induce negative feedback and a concomitant reduction in plasma Cort concentrations. Blood samples were taken 90 minutes following injection with DEX (DEXCort, calculated as [StressCort - corticosterone concentrations after DEX injection] to determine the strength of negative feedback; please note that due to this calculation positive values indicate strong negative feedback while negative values indicate that corticosterone concentrations after DEX injections were actually higher than after StressCort). Following this sample, we injected the individuals intramuscularly with a dose of 100 IU/kg of adrenocorticotropic hormone (ACTH; Sigma-Aldrich) to assess the maximal capacity of the adrenal gland to secrete corticosterone. A final blood sample was taken 30 min following ACTH injection (ACTHCort). Following this final blood sample, all birds were returned to their home cages. We determined plasma hormone concentrations using radioimmunoassays (see [77], following a double ether extraction of all samples (see Additional file 4 for more details including assay quality).

### Statistics

Body condition was calculated using the Scaled Mass Index [78]. Data were tested for normal distribution and homogeneity of variances using a combination of Kolmogorov-Smirnov and Levene tests as well as visual observations of histograms of the models’ residuals. Data were then transformed (as indicated in the Results section) appropriately before statistical analysis.

Short-term effects of the two treatment types on potential biomarkers were analysed separately (for detailed statistical methods and results see Additional file 4) and for reasons of divergent sample sizes and sampling times could not be included with analyses of long-term effects.

To analyse the long-term effects of the treatment, we compared repeated measurements of individuals from our two groups taken at the beginning with the ones taken at the end of the experiment. By analysing samples taken 1 year apart from each other we could eliminate possible seasonal variations) in telomeres, body mass, oxidative status, and stress hormones. We used Linear Mixed Models (LMMs, using random slopes and random intercepts if those improved the model fit) as described above, always including treatment, time, the interaction of treatment with ‘time’ as fixed factors and individual ID, as well as ID nested within nest ID as random variables. The variable ‘sex’ and the interactions of ‘sex*time’, ‘sex*group’ and ‘sex*time*group’ were first included but then removed from the models, as they did not explain a significant proportion of the variation.

All statistics were done in SPSS v 21.0 (Chicago). Data are publically available at the Dryad data repository (https://datadryad.org).

## Additional files

Additional file 1: Table S1. Efficacy of the immune challenge. Additional file 2: Table S2. Efficacy of the 10-day chronic disturbance protocol on stress hormones and oxidative stress load. Additional file 3: Table S3. Intraclass correlation coefficients (ICCs, [95% confidence intervals]) for all biomarkers. Additional file 4:
**General methodology.**
