# Supplementary material for: Repeated stressors in adulthood increase the rate of biological ageing
Source: Front Zool. 2015 Feb 13;12:4. doi: 10.1186/s12983-015-0095-z (PMC4336494; doi:10.1186/s12983-015-0095-z)
Supplement: Additional file 1: Table S1. — Efficacy of the immune challenge. [file 12983_2015_95_MOESM1_ESM.pdf]

Table S1: Efficacy of the immune challenge

Data were taken before and 24 hrs after injection of vehicle or LPS in Jan 2010. Data on IL-6-like bioactivity were collected 6 hrs after injection. Data are mean±1SEM, sample sizes given in brackets. * = effect of time (before-after, p<0.05).‡ effect of treatment*time interaction (p<0.05). Statistics are determined from LMMs (for details see text). – effects could not be determined; significant effects are highlighted in bold. # log-transformed data; ^ square-root-transformed data.

	**Control-before**	**Control-after**	**LPS injected-before**	**LPS injected-after**	Effect of treatment	Effect of time	Interaction treatment*time
**Il-6 bioactivity (absorbance at 595 nm)#**		0.03±0.01 (20)		0.076±0.011* (12)	**$F_{(1,32)}$= 43.63, p<0.0005**	-	-
**Body mass (g)**	85.28±2.27 (19)	82.89±2.2 (20)	86.83±1.94 (12)	80.01±1.77*‡ (12)	**$F_{(1,34.26)}$= 687.68, p<0.0005**	p>0.8	**$F_{(1, 34.26)}$=157.58, p<0.0005**
**Plasma oxidative damage (mM H_2O_2 equivalents)**	0.17±0.18 (20)	0.17±0.18 (19)	0.11±0.028 (7)	0.5±0.07*‡ (12)	**$F_{(1, 28.49)}$=11.12, p<0.002**	**$F_{(1, 23.24)}$=45.06, p<0.0005**	**$F_{(1, 23.24)}$=46.47, p<0.0005**
**Plasma non-enzymatic antioxidants (mM HOCl neutralized)#**	189.4±9.56 (20)	184.28±7.07 (20)	181.99±15.23 (7)	211.17±13.66 (11)	p>0.4	p>0.2	p>0.14
**Glutathione peroxidase (U/l hemolysate)^**	2320.69±112.13 (20)	2055.08±101.97 (20)	2645.07±230.07 (12)	2094.84±120.17* (12)	p>0.3	**$F_{(1, 32)}$= 24.44, p<0.0005**	p>0.14
