# Supplementary material for: Repeated stressors in adulthood increase the rate of biological ageing
Source: Front Zool. 2015 Feb 13;12:4. doi: 10.1186/s12983-015-0095-z (PMC4336494; doi:10.1186/s12983-015-0095-z)
Supplement: Additional file 2: Table S2. — Efficacy of the 10-day chronic disturbance protocol on stress hormones and oxidative stress load. [file 12983_2015_95_MOESM2_ESM.pdf]

Table S2: **Efficacy of the 10-day chronic disturbance protocol on stress hormones and oxidative stress load.**

A. Data for body mass and stress hormones were first collected in November 2009 and again right after the first 10-day chronic disturbance treatment in March 2010. B. Data for body mass and oxidative stress markers were collected before and after the last 10-day chronic disturbance treatment in November 2010. Data are mean±1SEM, sample sizes: control group (n=20), stress-exposed group (n=12). * = effect of time (before-after, p<0.05). ‡ = effect of treatment*time interaction (p<0.05). # = effect of treatment (p<0.05). $ = effect of sex (p<0.05). Statistics are determined from LMMs (for details see text). Significant effects are highlighted in bold. # log-transformed data; ^ square-root-transformed data.

**A.**	**Control group before**	**Control group after**	**Stress-exposed group before**	**Stress-exposed group after**	Effect of treatment	Effect of time	Interaction treatment*time
**Body mass (g)#**	89.59±2.28	85.56±2.52	85.88±1.65	83.98±2.27*	p>0.4	**$F_{(1,32)}$=6.94, p=0.013**	p>0.3
**Baseline corticosterone (ng/ml) #**	1.55±0.22	2.12±0.24	1.34±0.14	2.08±0.25*	p>0.8	**$F_{(1, 32)}$=11.11, p=0.002**	p>0.8
**Stress-induced corticosterone (ng/ml) ^**	11.97±2.31	25.58±4.63	9.15±2.2	14.76±2.84*	p=0.099	**$F_{(1, 32)}$=13.09, p=0.001**	p>0.2
**Difference between stress-induced corticosterone and concentrations after dexamethasone**	-2.64±2.64	-16.09±4.99	0.15±2.51	-10.29±2.68*	p>0.9	**$F_{(1,29.94)}$=11.65, p=0.002**	p>0.2

**injection (ng/ml)**							
**Corticosterone after ACTH-injection (ng/ml)**	40.53±4.61	50.31±4.23	43.01±6.03	59.19±8.33*[$]	p>0.4	**$F_{(1, 32)}$=6.24, p=0.018**	p>0.8
**B.**							
**Body mass (g) #**	90.79±2.63	88.22±2.43	86.4±1.55	81.88±1.25*‡	p>0.1	**$F_{(1, 32.56)}$= 50.56, p<0.0005**	**$F_{(1, 32.56)}$= 6.47, p=0.016**
**Plasma oxidative damage (mM of H_2O_2 equivalents) #**	0.11±0.013	0.14±0.01	0.4±0.14	0.17±0.02#‡	**$F_{(1, 25.17)}$= 5.79, p=0.024**	p>0.1	**$F_{(1,25.84)}$= 10.37, p=0.003**
**Plasma non-enzymatic antioxidants (mM HOCl neutralized) #**	204.59±7.23	203.63±7.6	230.28±11.86	242.74±11.01#	**$F_{(1,30.37)}$= 8.89, p=0.006**	p>0.3	p>0.2
**Glutathione peroxidase (U/l hemolysate) #**	2287.82±110.04	2289.85±93.75	2563.96±135.67	2494.91±133.31	p>0.1	p>0.1	$F_{(1, 32.15)}$= 2.99, p=0.093
