# Supplementary material for: Repeated stressors in adulthood increase the rate of biological ageing
Source: Front Zool. 2015 Feb 13;12:4. doi: 10.1186/s12983-015-0095-z (PMC4336494; doi:10.1186/s12983-015-0095-z)
Supplement: Additional file 3: Table S3. — Intraclass correlation coefficients (ICCs, [95% confidence intervals]) for all biomarkers. [file 12983_2015_95_MOESM3_ESM.pdf]

Table S3: **Intraclass correlation coefficients (ICCs, [95% confidence intervals])* for all biomarkers**

**ICCS:**	**Total (n=32)**	**Control group (n=20)**	**Stress-exposed group (n=12)**
Telomere length (bp)	**0.823** [0.512, 0.925] P<0.005*	**0.954** [0.843, 0.988] P<0.005*	**0.667** [0.008, 0.903) P=0.001*
Plasma oxidative damage (mM of H_2O_2 equivalents)	**0.174** [-0.093, 0.454] P=0.042*	**0.178** [-0.085, 0.525] P=0.01*	0.24 [-0.294, 0.689] P=0.198
Plasma non-enzymatic antioxidants (mM HOCl neutralized)	0.17 [-0.114, 0.455] P=0.12	0.109 [-0.191, 0.455] P=0.259	0.261 [-0.297, 0.704] P=0.186
Glutathione peroxidase (U/l hemolysate)	**0.246** [-0.099, 0.586] P=0.001*	**0.247** [-0.109, 0.604] P=0.011*	**0.21** [-0.081, 0.622] P=0.017*
Scaled mass index	**0.93** [0.859, 0.966] P<0.005*	**0.946** [0.87, 0.978] P<0.005*	**0.812** [0.488, 0.941] P<0.005*
Locomotor activity (hops/2min)	**0.652** [0.39, 0.851] P<0.005*	**0.636** [-0.002, 0.872] P<0.005*	**0.654** [0.175, 0.886] P=0.004*
Baseline corticosterone (ng/ml)	-0.011 [-0.353, 0.333] P=0.525	0.148 [-0.294, 0.542] P=0.259	-0.699 [-1.024, -0.09] P=0.987
Stress-induced corticosterone (ng/ml)	**0.518** [0.218, 0.73] P=0.001*	**0.542** [0.162, 0.786] P=0.004*	0.449 [-0.087, 0.797] P=0.054
Difference between stress-induced corticosterone and concentrations after dexamethasone injection (ng/ml) (ng/ml)	-0.019 [-0.287, 0.286] P=0.55	-0.045 [-0.408, 0.367] P=0.587	0.177 [-0.113, 0.58] P=0.126
Corticosterone after ACTH-injection (ng/ml)	0.132 [-0.179, 0.436] P=0.21	-0.115 [-0.509, 0.329] P=0.695	**0.625** [-0.006, 0.885] P=0.001*

*all biomarkers were log-transformed prior to analysis, using two-way mixed effects models with ‘birdID’ as random effect and ‘before – after’ measurements as fixed effect; in SPSS Version 21. Significant ICCs are highlighted in bold.
