# Supplementary material for: Repeated stressors in adulthood increase the rate of biological ageing
Source: Front Zool. 2015 Feb 13;12:4. doi: 10.1186/s12983-015-0095-z (PMC4336494; doi:10.1186/s12983-015-0095-z)
Supplement: Additional file 4: — General methodology. [file 12983_2015_95_MOESM4_ESM.pdf]

General methodology

Bird husbandry

Nestlings were collected from an urban (Munich; 48°07’ N, 11°34’ E, 518 m asl) and a nearby rural site (forest of Raisting 47°53’ N, 11°04’ E, 553 m asl; [1, 2]). All birds were banded with a unique aluminum rings. The birds used in this study were previously involved in behavioral and hormonal experiments [2]. However, we consider any carry-over effects from these experiments unlikely since experiments were primarily behavioral, and all birds were treated identically. Birds were kept in individual cages (HxWxD:75x45x80cm) in one of two rooms, and cages were equipped with passive motion sensors mounted to the top of each cage (Intellisense XJ-413T, by CK Systems, 12 m/40º range). Food (Granvit, Chemivit, Quattro Castella, Italy) and water were available *ad libitum* and both were renewed every day. All experimental procedures were conducted with prior approval by the appropriate authority, the Regierungspräsidium of Freiburg, Germany (permit number: 35/9185.81/G-08/97).

Blood sampling and sample processing

Blood samples were obtained by pricking the wing vein with a 23 gauge needle and collecting the blood samples in heparinized capillary tubes. Samples were kept on ice and were centrifuged within 4 hours for 10 minutes at 9000 g. Plasma was taken off and stored at -80ºC until analysis for hormone concentrations and oxidative stress markers. Red blood cells were added to a cryoprotectant buffer consisting of 90 % bovine serum and 10% DMSO for telomere analyses. Red blood cells from other sampling periods were frozen for later glutathione peroxidase analyses.

Lyophylized LPS (Sigma L2880) was reconstituted in phosphate-buffered saline (PBS) to a concentration of 1mg/ml. For each of the four LPS challenge periods all individuals from the stress-exposed group received an injection on the same day, always between 9-11 am. We injected a dose of 2.0 µg LPS diluted in PBS per gram body mass into the breast muscle using a 26g needle.

LPS injections

Lyophylized LPS (Sigma L2880) was reconstituted in phosphate-buffered saline (PBS) to a concentration of 1mg/ml. For each of the four LPS challenge periods all individuals from the stress-exposed group received an injection using a 26g needle into the breast muscle on the same day, always between 9-11 am.

Determination of cytokine concentrations

Plasma samples for IL-6 analyses were taken 6 hrs after the first LPS injection (in Jan 2010), in line with earlier research in songbirds suggesting that they would be elevated around this time [3]. IL-6-like bioactivity of plasma was determined via bioassay of cell proliferation in mouse B-cell hybridoma cells, using the methods of Van Oers et al. [4] with minor modification [3]). B9 cells, which are highly dependent upon IL-6 for growth, were maintained in RPMI media (cat. no. 11875-085 Gibco, Invitrogen, Carlsbad, CA, USA) supplemented with 5% fetal bovine serum (cat. no. SH3007002 Hyclone, Thermo Scientific, Waltham, MA, USA), 100 IU/ml penicillin/streptomycin (cat. no. SV30010, Hyclone), 50 µM 2-mercaptoethanol (cat. no. M6250, Sigma-

Aldrich), and 50 pg/ml recombinant human IL-6 (cat. no. I1395, Sigma-Aldrich, St. Louis, MO, USA). Prior to the assay, cells were washed twice in RPMI media containing no IL-6. Bird plasma samples were plated in duplicates of 10uL and then serially diluted from 1:2 to 1:64 in RPMI on 96-well tissue culture plates. B9 cells were added to each well at a density of 5,000 cells/well in RPMI media containing all of the above additives, except IL-6, and including a final concentration of 2.5x106 M of polymyxin B (cat. no. P1004, Sigma-Aldrich), which prevents direct activation of B9 cells by any potential LPS remaining in plasma samples. Cells were incubated and allowed to proliferate for 72hrs at 37°C, at which point 25µL of 5 mg/mL thiazolyl blue tetrazolium bromide dye (MTT, cat. no. M5655, Sigma-Aldrich) was added to every well. Four hours later, each well was treated with 50mL of a solution containing 20% SDS, 37.75% *N,N*-dimethyl formamide, 2% glacial acetic acid, 2.5% 1M HCl, and 37.75% de-ionized water. After incubating overnight at 37°C, cell proliferation was assayed by reading absorbance on a Bio-Rad iMark plate reader (cat. no. 168-1135, Life Science Research, Hercules, CA, USA). The final values reported reflect absorbance at 595 nm minus absorbance at 630 nm, which controls for differences in general opacity between wells.

To facilitate analysis similar to all other metrics in this study, an average absorbance was calculated across all dilutions of each sample, yielding a single value per sample. These values were then analyzed using mixed models as specified below. Analysis by nested mixed effects models, incorporating values of each serial dilution and autocorrelation functions, as in Adelman et al. [3], yielded qualitatively identical results.

Determination of telomere length

Briefly, DNA from whole blood was extracted in 0.8% agarose plugs, digested with proteinase K, followed by restriction digestion with 15 U of *Hinf*I, 75 U of *Hae*III, and 40 U of *Rsa*I at 37 °C. Plugs were loaded into a 0.8% nondenaturing agarose gel. DNA was separated using pulsed field gel electrophoresis (3 V/cm, 0.5–7.0 s switch times, 14 °C) for 21 h, followed by in-gel hybridization at 37 °C overnight with a radioactive-labeled telomere-specific oligo $(CCCTAA)_4$. Hybridized gels were placed on a phosphorscreen (Amersham Biosciences, Buckinghamshire, UK), which was scanned on a Storm 540 Variable Mode Imager (Amersham Biosciences). We used densitometry (ImageQuant 5.03v and ImageJ 1.42q) to determine the position and strength of the radioactive signal in each of the lanes compared to the molecular marker (1 kb DNA Extension Ladder; Invitrogen, CA, USA). The background was fixed as the nadir of the low MW region on the gel (<1 kb). Genome-wide mean telomere length was calculated in the range of 1-40 kb (the limits of our molecular marker). Intra- and inter-gel coefficients of variation were, respectively: 3.3% and 2.0%.

Oxidative status of blood

Plasma oxidative damage (mostly hydroperoxides) was measured by colorimetric determination using the d-ROMs test. The OXY-Adsorbent test was used to quantify the ability of plasma non-enzymatic antioxidant compounds to cope with the oxidant action of hypochlorous acid (HOCl; an endogenously produced oxidant). We chose this assay because compared to others (e.g., FRAP, TEAC) it does not overly rely on the contribution of uric acid. The concentration of glutathione peroxidase in red blood cells was quantified using the Ransel assay (Randox Laboratories, Crumlin, UK; for further details on all assays see [5]). Readings of absorbance were carried out with a Thermo

Scientific Multiskan Spectrum (ThermoFisher, Vantaa, Finland). All samples were run in duplicate and the respective intra- and inter-assay coefficients of variation were: 5.80 and 7.45% for hydroperoxides; 5.96 and 6.69% for plasma non-enzymatic antioxidant compounds; 5.65 and 8.05% for glutathione peroxidase levels.

Hormone analyses

Recovery of sample content after extraction was 84.8±5.4% (mean±CV). Corticosterone antibody was purchased from Esoterix Endocrinology, CA, USA. All samples were assayed in duplicates. A total of 8 assays were run and all samples from one individual collected during a particular sampling period were always included in the same assay. Two buffer blanks were taken through the entire assay procedure in each assay. Four samples with known amount of corticosterone added (at low, medium and high concentrations) were included in each assay to compute intra-assay (mean CV: 6.8%) and inter-assay variation (mean CV: 13.2%).

Statistics: Analyses of short-term effects of the two treatments on biomarkers

Short-term efficacy of LPS injection on body mass, cytokines (samples taken in Jan ’10) and on oxidative stress (samples taken in Oct ’10), and of chronic disturbance treatment on body mass, oxidative stress (samples taken in Nov ’10) and stress hormones (samples taken in Dec ’09 and Mar ’10) were analysed using linear mixed models in which we always included ‘treatment’ (vehicle versus LPS-injection, control: vehicle versus stress-exposed: LPS-injection), the interaction of treatment with ‘time’ (before versus after injection or before versus after stress exposure, respectively) as fixed effects and

individual ID, as well as ID nested within nest ID (to account for the fact that some individuals were siblings) as random factors. The variable ‘sex’ and the interactions of ‘sex*time’, ‘sex*group’ and ‘sex*time*group’ were included in the models (and will be reported in the Results section) if they increased the model fit and explained a significant proportion of the variation.

Results: Efficacy of the immune and chronic stress treatments used

Six hours after injection, plasma interleukin (IL)-6-like bioactivity was 2-fold higher in LPS-injected than in vehicle (PBS) injected birds (all raw data and statistical results are summarized in Table S1). Both groups of birds lost body mass following the injection, but LPS-injected birds lost significantly more body mass than PBS-injected individuals. LPS injected birds also showed a nearly 3-fold increase in plasma oxidative damage levels 24 hours after LPS injection, while PBS-injected individuals displayed consistently low oxidative damage levels. However, there were no differences between treatment groups, or changes over time in non-enzymatic antioxidant levels. Likewise, there were no group differences in glutathione peroxidase levels, although both groups decreased levels over time.

As a consequence of the chronic disturbance treatment, all birds lost body mass, but birds in the stress-exposed group decreased body mass more strongly than control birds (all raw data and statistical results are summarized in Table S2). Stress-exposed birds overall had higher plasma oxidative damage than control birds, and stress-exposed birds decreased while control birds increased oxidative damage during the chronic stress period. Stressed-exposed birds overall had higher plasma non-enzymatic antioxidant

levels, but levels did not vary over time, nor change differently in the two groups. There were no differences over time or between groups in blood cell glutathione peroxidase levels.

Following the 10-day repeated chronic stress disturbance regime body mass of all birds decreased over time (comparing Nov 09 to Mar 10), but groups did not differ. Although there were temporal changes in endocrine variables in both groups over time (BaseCort, StressCort and ACTHCort increased, whereas DEXCort decreased), there were no differences between groups nor interactions between time and treatment. For ACTHCort, there was a significant main effect of sex with females having higher concentrations of corticosterone than males (Table S2).

Discussion: Treatments caused short-term physiological challenges

Both of our stress treatments (i.e. immune challenge and chronic daily disturbances) were effective in delivering short-term stressful stimuli, in particular by decreasing body mass and affecting oxidative stress load (Tables S1, S2). Injections of LPS are known to cause robust immune responses in vertebrates by inducing the energetically costly acute phase immune response that can include fever, anorexia and sickness behaviour in birds [3, 6]. LPS is a protein expressed on bacterial cell walls, representing an ecologically relevant stimulus that individuals in the wild likely experience during their lives. As in a previous study on song sparrows [3], cytokine secretion of Eurasian blackbirds in our study was significantly increased within 6 hours after LPS-injection. Furthermore, corroborating previous studies showing that immune responses can induce oxidative damage and change antioxidant status (e.g., [7-9], within

24 hrs LPS-injected individuals in our study lost body mass and had increased levels of circulating oxidative damage (Table S1).

Likewise, birds exposed to 10 days of the chronic disturbance treatment lost mass (see also [10] and decreased plasma concentrations of oxidative compounds from an elevated level to that of control birds (Table S2). Surprisingly, effects of the chronic disturbance treatment on the endocrine stress axis were not detectable (Table S2). Our chronic disturbance protocol represented a milder version of that used previously in studies on European starlings (*Sturnus vulgaris*), in which more disturbances per day (up to 6) and for longer periods of time (up to about 3 weeks) resulted in reduced baseline and stress-induced corticosterone concentrations as well as a reduced maximal corticosterone secretion after ACTH injection [11, 12]. It is highly likely that in our experiments each individual disturbance on a given day induced an endocrine stress response, especially in the beginning of the treatment period [11], and that a longer duration of the disturbance regime may have resulted in an alteration of the endocrine stress axis. At the same time, the lack of significant changes in the endocrine stress axis in the current study suggests that the chronic disturbance treatment did not induce HPA pathology.

References

1. Partecke J, Schwabl I, Gwinner E: **Stress and the city: Urbanization and its effects on the stress physiology in European Blackbirds.** *Ecology* 2006, **87:**1945-1952.
2. Miranda AC, Schielzeth H, Sonntag T, Partecke J: **Urbanization and its effects on personality traits: a result of microevolution or phenotypic plasticity?** *Global Change Biology* 2013, **19:**2634-2644.

3. Adelman JS, Bentley GE, Wingfield JC, Martin LB, Hau M: **Population differences in fever and sickness behaviors in a wild passerine: a role for cytokines.** *Journal of Experimental Biology* 2010, **213:**4099-4109.
4. Van Oers MHJ, Van Der Heyden A, Aarden LA: **Interleukin 6 (Il-6) in serum and urine of renal-transplant recipients.** *Clinical and Experimental Immunology* 1988, **71:**314-319.
5. Costantini D, Monaghan P, Metcalfe NB: **Biochemical integration of blood redox state in captive zebra finches (Taeniopygia guttata).** *Journal of Experimental Biology* 2011, **214:**1148-1152.
6. Owen-Ashley NT, Hasselquist D, Raberg L, Wingfield JC: **Latitudinal variation of immune defense and sickness behavior in the white-crowned sparrow (Zonotrichia leucophrys).** *Brain Behavior and Immunity* 2008, **22:**614-625.
7. Sirak AA, Beavis AJ, Robertson FM: **Enhanced hydroperoxide production by peripheral blood leucoyctes following exposure of murine epidermis to 12-O-tetradecanoylphorbol-13-acetate. .** *Carcinogenesis* 1991, **12:**91-95.
8. Costantini D, Møller AP: **Does immune response cause oxidative stress in birds? A meta-analysis.** *Comparative Biochemistry and Physiology - Part A: Molecular & Integrative Physiology* 2009, **153:**339-344.
9. van de Crommenacker J, Horrocks NPC, Versteegh MA, Komdeur J, Tieleman BI, Matson KD: **Effects of immune supplementation and immune challenge on oxidative status and physiology in a model bird: implications for ecologists.** *Journal of Experimental Biology* 2010, **213:**3527-3535.
10. Cyr NE, Earle K, Tam C, Romero LM: **The effect of chronic psychological stress on corticosterone, plasma metabolites, and immune responsiveness in European starlings.** *General and Comparative Endocrinology* 2007, **154:**59-66.
11. Rich EL, Romero LM: **Exposure to chronic stress downregulates corticosterone responses to acute stressors.** *Am J Physiol Regul Integr Comp Physiol* 2005, **288:**R1628-1636.
12. Dickens MJ, Earle KA, Romero LM: **Initial transference of wild birds to captivity alters stress physiology.** *General and Comparative Endocrinology* 2009, **160:**76-83.
